# Role of Bmi-1 in Regulation of Ionizing Irradiation-Induced Epithelial-Mesenchymal Transition and Migration of Breast Cancer Cells

**DOI:** 10.1371/journal.pone.0118799

**Published:** 2015-03-03

**Authors:** Weiwei Yuan, Ye Yuan, Tao Zhang, Shiyong Wu

**Affiliations:** 1 Department of Oncology, the General Hospital of Chengdu Military District, Chengdu, Sichuan, P. R. China; 2 Department of Chemistry and Biochemistry, and Edison Biotechnology Institute, Ohio University, Athens, Ohio, 45701, United States of America; University of Kentucky, UNITED STATES

## Abstract

Radiotherapy is a widely used treatment for cancer. However, recent studies suggest that ionizing radiation (IR) can promote tumor invasion and metastasis. Bmi-1, a member of the polycomb group protein family, has been observed as a regulator of oxidative stress and promotes metastasis in some tumors. But, its potential role in the metastasis induced by IR of breast cancer has not been explored. In our study, we found that increased levels of Bmi-1 were correlated to EMT of breast cancer cells. Through analyzing the EMT state and metastasis of breast cancer induced by IR, we found the metastatic potential of breast cancer cells can either be inhibited or accelerated by IR following a time-dependent pattern. Silencing Bmi-1 completely abolished the ability of the IR to alter, reduce or increase, the migration of breast cancer cells. Also, when Bmi-1 was knocked down, the effect of inhibition of PI3K/AKT signaling on EMT affected by IR was blocked. These results suggest that Bmi-1 is a key gene in regulation of EMT and migration of breast cancer cells induced by IR through activation of PI3K/AKT signaling; therefore, Bmi-1 could be a new target for inhibiting metastasis caused by IR.

## Introduction

Ionizing radiation (IR) is a widely used therapeutic modality for various human tumors, including breast cancer. It triggers the production of reactive oxygen species, which damage the DNA and induce apoptosis or senescence of cancer cells [1,2]. However, several recent studies observed that radiotherapy have certain unpredictable effects on epithelial–mesenchymal transition (EMT), which plays a major role in the invasion or metastasis of cancer cells [3,4]. It was reported that in lung cancer cells, high does of IR induced a series of EMT-associated changes via p38-MAPK signaling pathway at 48 h after IR [5]. Meanwhile, IR promoted EMT and metastasis of lung cancer cells and colorectal adenocarcinoma cells through activation of TGF-β signaling at 5–7 days after IR [6]. While these reports suggested IR might promote metastatic potential of cancer cells, the molecular mechanisms by which IR promotes cancer cell metastasis have not been fully elucidated.

Bmi-1 (B-cell-specific Moloney murine leukemia virus insertion site 1) is identified as an oncogene which is a member of the polycomb group protein family [7,8]. Bmi-1 is important for the self-renewal of both normal and cancer stem cells, which is overexpressed in various tumors, such as breast cancer, lung cancer, colorectal cancer, prostate cancer and hepatocellular cancer [9–13]. Its over-expression accelerates oncogenic transformation and metastatic potential of cancer cells. A new role of Bmi-1 in mitochondrial function was investigated, suggesting that Bmi-1 was involved in regulation of the oxidative stress levels by suppressing the expression of oxidase genes [14–16]. In this report, we investigated the effect of IR on the expression of Bmi-1 and its effect on EMT and metastasis of breast cancer cells in a time-dependent manner. Our results indicated that Bmi-1 might be a key gene in regulation of IR-altered breast cancer metastatic potential.

## Materials and Methods

### Cells culture and construction of Bmi-1 stably transfected cell lines

Human breast cancer cell lines (MDA-MB-468, MDA-MB-231, T-47D, Hs578t, MCF-7) were purchased from the American Type Culture Collection (ATCC, Manassas, VA). All the breast cancer cells were grown in Dulbecco’s Modified of Eagle’s Medium supplemented with 10% fetal bovine serum (FBS, Invitrogen, San Diego, CA). All cells were kept under 95% air and 5% CO_2_ at 37°C.

Hs578t and MDA-MB-231cells were transfected with Bmi-1 small hairpin RNA (shRNA, Santa Cruz, CA) and non-target vectors (NC, negative control) at an 80% to 90% confluence using Lipofectamine 2000 transfection reagent (Invitrogen, San Diego, CA) according to manufacturer’s protocol. For stable transfection, cells were passaged at 1:10 into fresh growth medium 24 h after transfection. G418 was added at a final concentration of 1200 μg/mL for MDA-MB-231 cells and 600 μg/mL for Hs578t cells during selection, and 600 μg/mL for MDA-MB-231 cells and 300 μg/mL for Hs578t for maintenance of the transfected cells. The efficiency of Bmi-1 inhibition was determined by Western blot analysis.

### Chemical and IR treatments

Phosphoinositide 3-kinase(PI3K) inhibitor (LY294002) and Akt 1/2 kinase inhibitor (Akt I) were purchased from Sigma–Aldrich (St. Louis, MO). The final concentrations for the treatment were 5 μM LY294002 and 2 μM Akt I. The cells were pre-treated with each chemical for 1 h and then exposed to a γ–ray source at a dose rate of 1 Gy/min, for a total dose of 2 Gy for each treatment. After the irradiation, the cells were incubated for 18 h in the same medium with each inhibitor.

### Western blot analysis

The antibodies against E-cadherin, Vimentin, and p-Akt were purchased from Cell Signaling Technology (Danvers, MA). The antibodies for Akt and β-actin were purchased from Santa Cruz Technology (Santa Cruz, CA). The antibody against Bmi-1 was purchased from Millipore (Bedford, MA). Cells were washed twice with ice-cold PBS, and then lysed with lysis buffer (100 mM Tris-HCl pH 8.0, 80 mM NaCl, 2% NP-40, 0.1% SDS) containing Proteinase Inhibitor. Then, lysates were subjected to SDS-PAGE with a 10% SDS polyacrylamide gel and transferred to a nitrocellulose membrane, which was then blocked with 5% (w/v) skim milk in TBST (20 mM Tris-HCl, pH 7.5, 150 mM NaCl, 0.1% Tween 20) for 1 h and then incubated with primary antibodies at 4°C overnight. After washing with TBST, the membrane was incubated with HRP–conjugated secondary. The membrane was then washed three times in TBST and developed in West Pico Supersignal chemiluminescent substrate (Pierce, Rockford, IL).

### Migration assay

Cells were plated in the upper chambers (8 μm, 24-well) containing 200 μL medium with 1% FBS. 800 μL medium with 10% FBS was injected into the lower chamber. After 24 h, non-migrating cells were removed from the upper chambers and the membranes were fixed with 4% formaldehyde. Then the migratory cells that were attached on the undersurface of chamber were stained with 1% crystal violet solution. After being washed with PBS and dried, the numbers of invaded cells were counted by microscope at 5 different fields.

### Flow cytometry

The apoptotic death of cells was analyzed using a fluorescein isothiocyanate (FITC) conjugated-annexin V (ANX5)/propidium iodide (PI) apoptosis detection kit (BD Biosciences, Franklin Lakes, NJ) following the manufacturer’s protocol. In brief, the cells were harvested, combined with the cells floating in the medium and washed the cells twice with cold PBS solution. The cells were then suspended in ANX5 binding buffer to 10^6^ cells/mL and 100 μL of the cell suspension was mixed with 5 μL ANX5-FITC and 5 μL PI. The cell mixture was incubated in dark for 15 min and the ANX5/PI double-stained cells were analyzed by using a FACSort Flow Cytometer (BD science, Franklin Lakes, NJ) equipped with CellQuest software (BD science, Franklin Lakes, NJ). The numbers of cells positive for both ANX5 and PI were counted.

### Statistical analysis

Statistical analysis was performed using the SPSS 18.0 software package. Values were expressed as mean ± SD of three independent experiments. The data in the different treatment groups were compared by independent samples t-test. P-value less than 0.05 was considered statistically significant.

## Results

### Expression level of Bmi-1 is correlated to EMT of breast cancer cells

**Fig 1:**
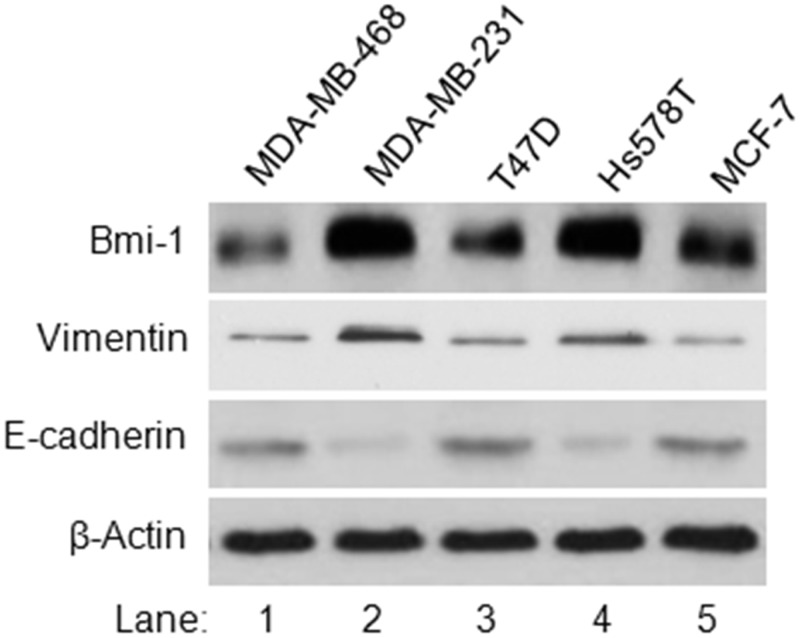
Analysis of the expressions of Bmi-1 and EMT makers in five breast cancer cell lines. The protein expression levels of Bmi-1, vimentin, E-cadherin and β-action were determined using Western blot analysis.

To determine the potential role of Bmi-1 in regulation of EMT of breast cancer cells, we determined whether there is a correlation between the expression of Bmi-1 protein with EMT markers in five breast cancer cell lines. Our data indicated while Bmi-1 protein was detected in all the tested breast cancer cell lines, its expression level was higher in MDA-MB-231 and Hs578t cells than the others (Fig. 1). Further analysis of EMT markers revealed that the increased expressions of Bmi-1 in breast cancer cell lines were correlated to increased expression of vimentin and decreased expression of E-cadherin (Fig. 1). As vimentin elevation and E-cadherin repression are markers of EMT, our results suggested that Bmi-1 might play a role in regulation of EMT of breast cancer cells.

### IR-regulated expression of Bmi-1 and migration of breast cancer cells are time-dependent

**Fig 2:**
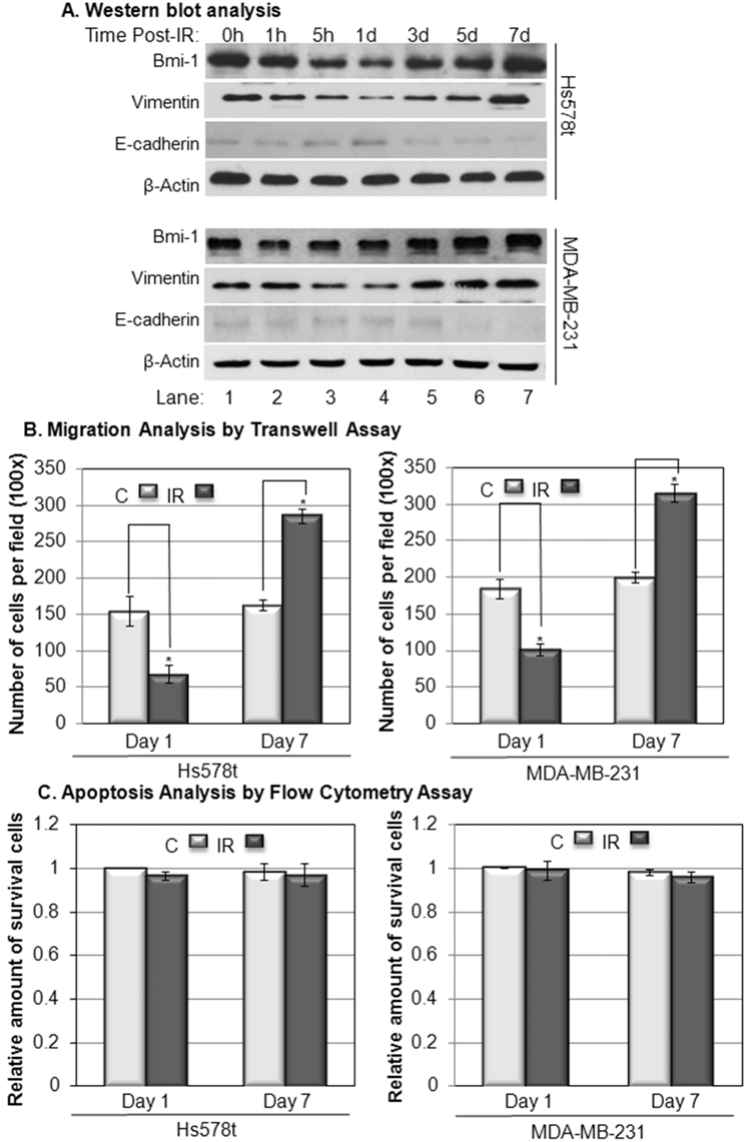
The time-dependent analysis of Bmi-1 expression and its correlation with migration of Hs578t and MDA-MB-231 cells after IR. Panel A: The cells were treated with IR (2 Gy) and lysed at indicated time-points. The protein expression levels in total lysates were determined using Western blot analysis. Panel B: Migrations of the cells were determined by the transwell assay at day 1 (24 h) and day 7 (168 h) post-IR (2 Gy). Panel C: Apoptosis of the cells was analyzed by flow cytometry at day 1 and day 7 post-IR (2 Gy). The data represents the means ± SD from 3 independent experiments. *: *p*<0.05.

Our previous data indicated that IR induces vimentin expression [17]. To determine the potential role of Bmi-1 in regulation of IR-induced vimentin expression and EMT of breast cancer cells, we analyzed the correlation of the expression levels of Bmi-1 with vimentin and E-cadherin in MDA-MB-231 and Hs578t cells after IR (2 Gy) in a time-dependent manner. Our data indicated that Bmi-1 in Hs578t cells was decreased and reached its lowest point at one day post-IR (Fig. 2A, top panel, lane 4), and then gradually recovered to a level that was higher than background at seven days post-IR (Fig. 2, top panel, lane 7). The expression levels of vimentin and E-cadherin were positively and negatively correlated, respectively, to the expression level of Bmi-1 post-IR (Fig. 2A, top panel). The expressions of these proteins in response to IR in MDA-MB-231 cells had a similar trend as they did in Hs578t cells (Fig. 2A, bottom panel). These results suggest that IR has an inhibitory effect on metastatic potential of breast cancer cells at early phase (1 day), but has an opposite effect at late phase (7 days) post-IR.

To confirm this hypothesis, the migration of Hs578t and MDA-MB-231 cells at the 1st and 7th day after IR (2 Gy) were determined using a transwell assay. Our data indicated that the migration of the breast cancer cells was reduced at day one but increased at day 7 post-IR (Fig. 2B). The decreased migration of breast cancer cells was not due to the potential effect of cell apoptosis caused by IR because the amount of apoptotic cells was not statistically significantly change at either day 1 or day 7 after the treatment (Fig. 2C). These results indicated that IR can either inhibit or accelerate migration of breast cancer cells following a time-dependent pattern, and Bmi-1 may be involved in this process.

### IR-induced EMT process and migration of breast cancer cells are Bmi-1 dependent

**Fig 3:**
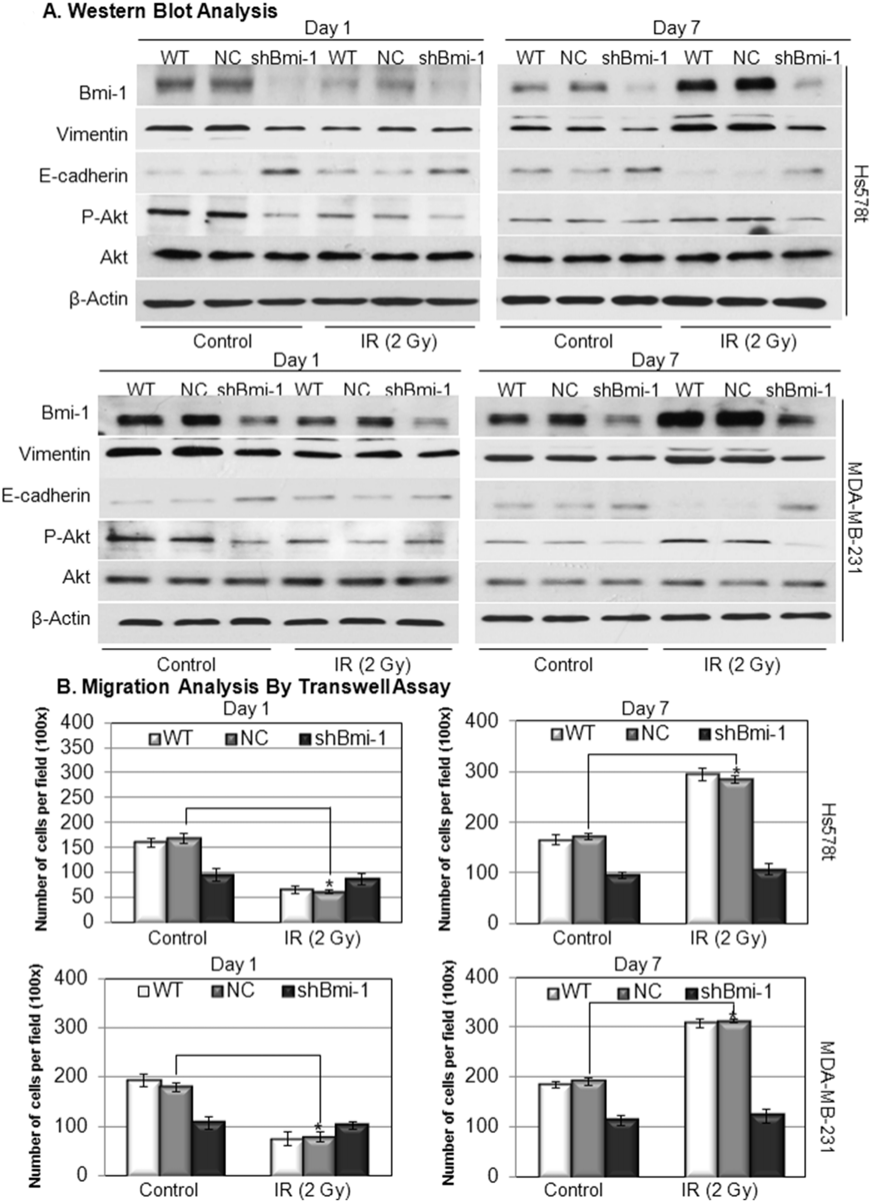
The effect of Bmi-1 knockdown on IR-altered EMT process and migration of Hs578t and MDA-MB-231 cells. Panel A: Established Hs578t and MDA-MB-231 cell lines infected with Bmi-1-targeting shRNA (shBmi-1) or non-targeting control shRNA (NC) and wild type (WT) cells were treated with IR (2 Gy) and lysed at day 1 (24 h) and day 7 (168 h) post-IR (2 Gy). The protein expression levels in total lysates were determined using western blotting analysis. Panel B: Migrations of the cells were determined by the transwell assay at day 1 and day 7 post-IR (2 Gy). The data represents the means ± SD from 3 independent experiments. *: *p*<0.05.

To further confirm the role of Bmi-1 in regulation of metastatic potential of breast cancer cells after IR, Bmi-1 expression in Hs578t and MDA-MB-231 cells was knocked down using lentivirus encoding Bmi-1-targeting shRNA. The knockdown efficiency and its effect on the expressions of EMT markers (vimentin and E-cadherin) were determined by western blot analysis. Our data indicate that Bmi-1 expression is significantly reduced in the cells infected with Bmi-1-targeting virus at day 1 and 7 post-IR (Fig. 3A, shBmi-1 vs. NC or WT); and the reduction of Bmi-1 expression is correlated with a reduced vimentin and increased E-cadherin expression (Fig. 3A, shBmi-1 vs. NC or WT). Furthermore, the knockdown of Bmi-1 completely abolished the ability of the IR to alter, reduce or increase, the migration of breast cancer cells (Fig. 3B, shBmi-1 vs. NC or WT). These results indicate that Bmi-1 is a key regulator of the EMT process and migration of breast cancer cells after IR.

### Bmi-1 regulate IR induced EMT and migration of breast cancer cells through activation of PI3K/AKT signaling pathway

**Fig 4:**
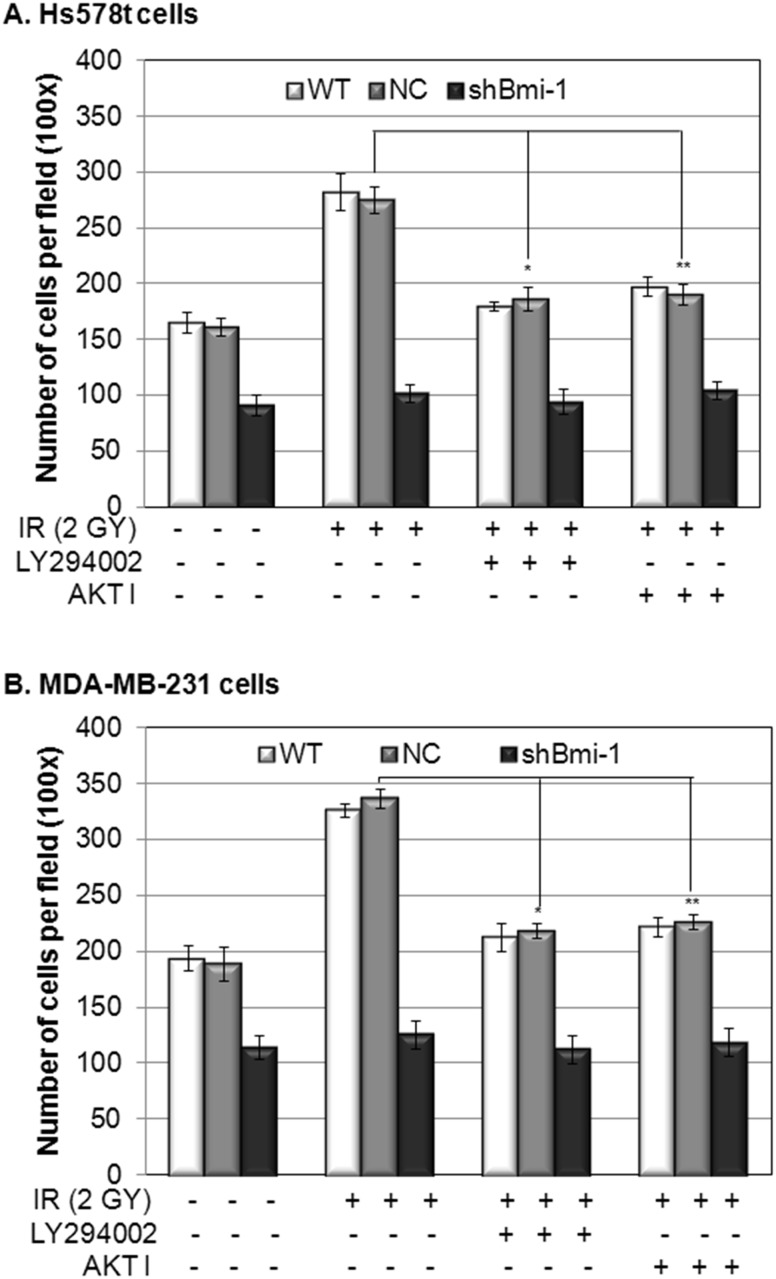
The effect of PI3K and Akt inhibitors on IR-altered EMT process and migration of breast cancer cells and breast cancer cells with Bmi-1 knockdown. Established Hs578t (Panel A) and MDA-MB-231 (Panel B) cell lines infected with Bmi-1-targeting shRNA (shBmi-1) or non-targeting control shRNA (NC) and wild type (WT) cells were treated with IR (2 Gy) in the presence or absence of LY294002 or AKT I. Migrations of the cells were determined by the transwell assay at day 7 after the treatment. The data (A–B) represents the means ± SD from 3 independent experiments. *: p<0.05, IR+ LY294002 vs. IR alone; **: p<0.05 IR+AKT I vs. IR alone.

Bmi-1 has been shown to be an upstream regulator of the PI3K/Akt pathway [13,18,19], which plays a critical role in regulation of the EMT process and cell migration. Our data shows that Bmi-1 knockdown is correlated to reduced phosphorylation of Akt after the IR treatment (Fig. 3A, shBmi-1 vs. NC or WT). To determine whether Akt mediates IR-induced Bmi-1-regulated migration of breast cancer cells, we analyzed the effect of PI3K inhibitor LY294002 or AKT 1/2 kinase inhibitor AKT I on the migration of the same set of breast cancer cell lines after IR. Our data demonstrated that while LY294002 or AKT I inhibited the induced migration of the wild-type (WT) and the non-targeting lentivirus infected (NC) breast cancer cells at day 7 post-IR, neither LY294002 nor AKT I affected the migration of Bmi-1 knockdown (shBmi-1) breast cancer cells (Fig. 4, shBmi-1 vs. NC or WT). These results indicate that Bmi-1 regulates IR-induced EMT and migration of breast cancer cells through activation of PI3K/AKT signaling.

## Discussion

During the past century, radiation therapy has been used extensively as a curative or adjuvant cancer treatment, and radiation as a palliative measure for patients with advanced cancer [20,21]. However, some recent studies have indicated that IR may have pro-metastatic effects [22–25], but the mechanism and key genes that are involved in radiotherapy-associated side effects have not all been elucidated. The conclusion regarding the role of IR in regulation of cancer metastasis still remains controversial [3]. In this study, we demonstrated that IR regulates EMT process and migration of breast cancer cells in a time-dependent manner (Fig. 2). The reduction in the early phase (1 day) and induction in the late phase (2–7 days) of EMT process and migration of breast cancer cells post-IR are not only correlated to the expression levels of Bmi-1 post-IR, but also dependent on the expression of Bmi-1 (Figs. 2–3). Knockdown of Bmi-1 using Bmi-1-targeting shRNA totally abolished the effect of the IR on the EMT process and migration of breast cancer cells (Fig. 3). Besides Bmi-1, the PI3K/Akt cascade is also involved in regulation of the IR-induced EMT process and migration of breast cancer cells demonstrated by the results showing that either PI3K inhibitor or Akt inhibitor can abolish the inducibility of the effect of IR on breast cancer cells (Fig. 4). Interestingly, while the PI3K and Akt inhibitors can only suppress the IR-induced EMT process and migration of breast cancer cells, knockdown Bmi-1 inhibits the behavior of breast cancer cells with or without IR (Fig. 4). Furthermore, the PI3K and Akt inhibitors have no effect on the breast cancer cells with Bmi-1 knockdown (Fig. 4). These results indicate that Bmi-1 contributes the IR-induced EMT process and migration of breast cancer cells via PI3K/Akt pathway; and suggest that Bmi-1 could be a potential target for suppression of metastatic potential of breast cancer cells with or without IR.
